# The Differentiation of Human Cytomegalovirus Infected-Monocytes Is Required for Viral Replication

**DOI:** 10.3389/fcimb.2020.00368

**Published:** 2020-07-31

**Authors:** Chan-Ki Min, Akhalesh K. Shakya, Byeong-Jae Lee, Daniel N. Streblow, Patrizia Caposio, Andrew D. Yurochko

**Affiliations:** ^1^Department of Microbiology and Immunology, Center for Molecular and Tumor Virology, Louisiana State University Health Sciences Center-Shreveport, Shreveport, LA, United States; ^2^Vaccine and Gene Therapy Institute, Oregon Health and Science University, Beaverton, OR, United States; ^3^Center for Cardiovascular Diseases and Sciences, Louisiana State University Health Sciences Center-Shreveport, Shreveport, LA, United States; ^4^Feist-Weiller Cancer Center, Louisiana State University Health Sciences Center-Shreveport, Shreveport, LA, United States; ^5^Center of Excellence in Arthritis and Rheumatology, Louisiana State University Health Sciences Center-Shreveport, Shreveport, LA, United States

**Keywords:** HCMV, monocyte, macrophage, EGFR, integrin, signaling, differentiation, macrophage polarization

## Abstract

Viral dissemination is a key mechanism responsible for persistence and disease following human cytomegalovirus (HCMV) infection. Monocytes play a pivotal role in viral dissemination to organ tissue during primary infection and following reactivation from latency. For example, during primary infection, infected monocytes migrate into tissues and differentiate into macrophages, which then become a source of viral replication. In addition, because differentiated macrophages can survive for months to years, they provide a potential persistent infection source in various organ systems. We broadly note that there are three phases to infection and differentiation of HCMV-infected monocytes: (1) Virus enters and traffics to the nucleus through a virus receptor ligand engagement event that activates a unique signalsome that initiates the monocyte-to-macrophage differentiation process. (2) Following initial infection, HCMV undergoes a “quiescence-like state” in monocytes lasting for several weeks and promotes monocyte differentiation into macrophages. While, the initial event is triggered by the receptor-ligand engagement, the long-term cellular activation is maintained by chronic viral-mediated signaling events. (3) Once HCMV infected monocytes differentiate into macrophages, the expression of immediate early viral (IE) genes is detectable, followed by viral replication and long term infectious viral particles release. Herein, we review the detailed mechanisms of each phase during infection and differentiation into macrophages and discuss the biological significance of the differentiation of monocytes in the pathogenesis of HCMV.

## Introduction

HCMV is a global infectious pathogen with 56–94% seroprevalence in adults worldwide (Zuhair et al., 2019). The reported seroprevalence varies depending on the economic status of infected individuals and the country in which the individual resides in. The severity of clinical symptoms caused by HCMV infection is associated with the immunological status of the host. In most cases, primary infection in immunocompetent individuals results in mild or no symptoms; however, HCMV can cause the development of mononucleosis in some individuals. In cases of maternal primary infection or reinfection during pregnancy, HCMV can cross the placenta and result in severe fetal complications such as hearing loss and microcephaly (Yamamoto et al., 2011; Gabrielli et al., 2012; Lanzieri et al., 2017; Britt, 2018). In immune-compromised individuals, including AIDS patients and solid organ/bone marrow transplant recipients, HCMV infection can cause severe morbidity and mortality (Boehme et al., 2006; Ramanan and Razonable, 2013; Adland et al., 2015; Stern et al., 2019). For example, HCMV infection can cause pneumonia, retinitis, encephalitis, and bowel disease (Arribas et al., 1996; Boeckh et al., 2003; Heiden et al., 2007; Garrido et al., 2013; Fonseca Brito et al., 2019). In organ transplant recipients, HCMV-mediated disease and the risk for the development of HCMV-mediated disease is dependent on the nature of the transplant (i.e., solid organ vs. bone marrow; Ramanan and Razonable, 2013; Stern et al., 2019). In addition, chronic reactivation and long-term infection also appears to result in various cardiovascular diseases including atherosclerosis and restenosis (Zhou et al., 1996; Gilbert and Boivin, 2005).

Blood-borne monocytes, derived from CD34^+^ HPC in the bone marrow, play a pivotal role in replenishment of tissue resident macrophages involved in surveillance and elimination of foreign pathogens. Under normal condition, these short lived cells circulate in the bloodstream and only infiltrate into secondary lymphoid organs and diverse tissues when recruited via cytokines or other signaling processes, which in turn allows their interaction with endothelial cells and their subsequent migration through the endothelium (Serbina et al., 2008; Jakubzick et al., 2013, 2017; Varol et al., 2015). However, upon pathogenic challenge, monocytes can rapidly migrate to sites of infection and differentiate into macrophages or dendritic cells (DCs). These activated and differentiated innate immune cells can initiate systemic immune responses directed toward pathogens via secretion of inflammatory cytokines and delivery of antigen to secondary lymphoid organs (Hume et al., 2019). Due to their biopotency and immunological function, diverse pathogens specifically target monocytes in order to evade and manipulate systemic immune responses (Nikitina et al., 2018). In addition, monocytes show a strong capacity to migrate to nearly all tissues in the body as part of their homeostatic and immune functions, which can be manipulated by pathogens during infection to promote wide dissemination of the infectious agent harbored in these infected monocytes.

During primary infection and following reactivation, HCMV replication in monocytes/macrophages is tightly associated with differentiation of infected monocytes into macrophages (Taylor-Wiedeman et al., 1991, 1994; Maciejewski et al., 1993; Mendelson et al., 1996; Smith et al., 2004a; Chan et al., 2012; Stevenson et al., 2014). For example, in monocytes isolated from HCMV sero-positive individuals, 0.01% of monocytes contain the viral genome but showed undetectable levels of viral gene expression. However, treatment of monocytes with hydrocortisone or PMA induced differentiation into macrophages as well as HCMV IE gene expression (Taylor-Wiedeman et al., 1994). In addition, treatment with granulocyte-colony stimulating factor (G-CSF) induces HCMV reactivation in human latently infected monocytes/macrophages in a humanized animal model (Smith et al., 2010). Primary infection of monocytes with HCMV also induces differentiation of monocytes into macrophages; these virus-differentiated macrophages show a macrophage phenotype with expression of key macrophage markers (Smith et al., 2004a, 2010; Noriega et al., 2014). At 3 weeks post-infection, these differentiated macrophages begin to produce immediate early (IE) proteins, which triggers the early (E) and late (L) gene expression that initiates the production of mature virus (Ibanez et al., 1991; Smith et al., 2004a; Stevenson et al., 2014). In addition, the mobility and function of these differentiated monocytes/macrophages is significantly increased (Ibanez et al., 1991; Smith et al., 2004a, 2007; Chan et al., 2008, 2009a; Stevenson et al., 2014; Collins-McMillen et al., 2017).

### Monocytes and Macrophages

Mononuclear phagocytic cells including monocytes, macrophages, and DCs—are associated with normal homeostatic properties in the body, in addition to their role in immunological surveillance (Arandjelovic and Ravichandran, 2015; Rodero et al., 2015; Yona and Gordon, 2015). Monocytes, circulating in the blood, have a short half-life (~1.6 day) and can respond to a variety of pathogens, which results in their activation and the initiation of innate and adaptive immune responses (Patel et al., 2017; Hume et al., 2019). Following pathogenic stimulation, monocytes can differentiate into macrophages and/or DCs. While DCs deliver antigens to secondary lymphoid organs to stimulate naïve lymphoid cells such as T and B cells, macrophages can trigger local inflammatory responses and show a heightened phagocytic ability for a variety of pathogens. These differentiated monocytes/macrophages can also replenish tissue resident macrophages that originated from fetal liver and the yok sac (Jakubzick et al., 2017). The nature of the signaling that controls differentiation is complex with many receptors and soluble factors controlling the process. For example, differentiation can be initiated by pattern recognition receptors (PRRs) and independent of this process, through a variety of cytokines and other stimuli (Goudot et al., 2017).

### The Infinite Loop of HCMV Infection

**Figure 1:**
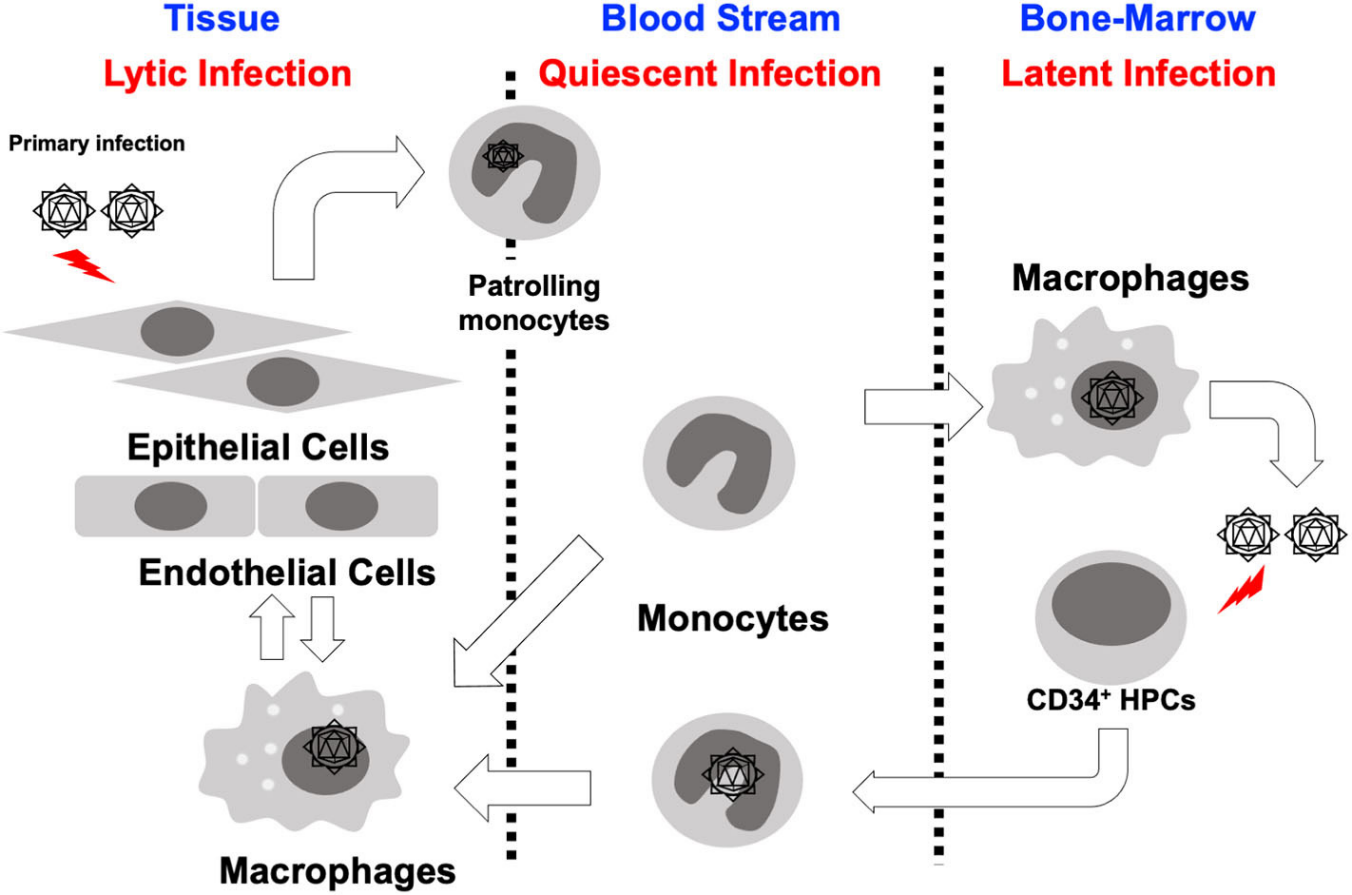
Cycle of HCMV infection, latency and reactivation. HCMV infected epithelial cells at the initial infection site likely rapidly produce virus, which spreads to adjacent cells at these local sites of infection. Monocytes infected with HCMV either as they are patrolling, or perhaps by free virus, then infiltrate into a variety of organ tissues and spread virus throughout the whole body. These monocytes differentiate into macrophages allowing for organ persistence and infection of new epithelial cells and virus release in various bodily fluids. Some of these infected monocytes infiltrate into the bone marrow and allow the establishment of latency in CD34^+^ HPCs. During a reactivation event, monocytes develop from CD34^+^ HPCs spreading virus throughout the whole body, again.

Long-term maintenance of HCMV in a host is the result of the careful orchestration of the process of latency and reactivation in infected hematopoietic progenitor cells. HCMV has a diverse host cell tropism and virus is produced with different rates depending on the type of infected cell (Collins-McMillen et al., 2018a). In fibroblasts and endothelial/epithelial cells viral particles are produced via a lytic replication cycle, but in CD34^+^ hematopoietic stem cells (HPCs), the virus establishes a latent infection (Sinzger et al., 2008; Goodrum, 2016; Collins-McMillen et al., 2018a). In contrast, it seems that infection of monocytes serves as a key bridge between lytic and latent phases of infection (Figure 1; Smith et al., 2004a; Chan et al., 2012). That is to say that monocytes are a key cell type for both lytic and latent processes. At the site of initial infection, HCMV first infects epithelial cells where the virus begins to proliferate and spread to adjacent cells. It appears that the virus spreads via a cell-to cell-mediated route rather than a cell-free route since virus is usually undetectable in the blood stream (Sinzger and Jahn, 1996). Monocytes then become the next target of the virus. Infected monocytes are a vector for viral spread due to their mobility and ability to migrate into most organ tissues (Sinzger and Jahn, 1996; Smith et al., 2004a), and it is the infiltration of infected monocytes into the bone marrow that is required for the establishment of latent infection in the CD34^+^ HPC reservoir (Streblow and Nelson, 2003; Wills et al., 2015; Collins-McMillen et al., 2018a). During reactivation, HCMV infected CD34^+^ HPCs can develop into monocytes allowing viral spread throughout the body (Smith et al., 2010; Crawford et al., 2018; Zhu et al., 2018). This cycle is repeated to maintain HCMV in infected individuals and in the human population as a whole; thus, there exists an exquisite balance between reactivation and latency. This model suggests that monocytes play a central role in the dissemination and pathogenesis of HCMV.

### Polarization of HCMV-Infected Monocytes

Due to the multi-potency and motility of monocytes, many pathogens target monocytes as reservoirs of infection (Nikitina et al., 2018). Many studies on the differentiation of monocytes discuss the process in terms of immune evasion and/or dissemination of the infecting pathogen (Hou et al., 2012; Foo et al., 2017; Ayala-Nunez et al., 2019). For example, Asian-lineage Zika virus (ZIKV) polarizes infected monocytes toward M2 macrophages that express anti-inflammatory cytokines such as IL-10 that likely suppress the adaptive immune response in pregnant women (Foo et al., 2017). In addition, hepatitis B core antigen triggers M2 polarization of monocytes via TLR2-singaling pathway (Yi et al., 2020). In line with these observations, we have documented that HCMV polarizes monocyte differentiation into inflammatory macrophages (Chan et al., 2009a; Stevenson et al., 2014) with a specific bias toward an M1 phenotype. However, others have shown that HCMV infection can inhibit the full differentiation of infected monocytes induced to differentiate with cytokines generated during allogeneic immune responses (Gredmark et al., 2004a,b). It was also shown that HCMV infection can block monocyte differentiation into DCs, likely through secretion of anti-inflammatory cytokines such as IL-10 (Gredmark and Soderberg-Naucler, 2003). Results from our transcriptome analysis supported HCMV-induced differentiation of monocytes into macrophages and specifically showed that ~70% of upregulated genes in HCMV-infected monocytes are considered M1 or M2 macrophage transcripts. Since the transcript pattern had a greater number of proinflammatory transcripts we favor labeling of infected monocytes as having a biased polarization toward an M1 phenotype (Chan et al., 2008, 2009a; Stevenson et al., 2014). We realize that there are M2 cytokines secreted from infected cells, so it seems that, although biased toward M1, they likely have a blended unique phenotype that favors viral replication. So why might there be differences with HCMV-mediated differentiation? Besides being different systems, the data provides biological clues to disease. Perhaps the block in differentiation due to cytokines produced during alloreactivity explain HCMV disease in allogenic stem cell transplant patients (Gredmark et al., 2004b). Collectively, the data suggest HCMV regulates the polarization and differentiation of monocytes towards a unique M1/M2 macrophage that favors viral persistence under normal infection conditions.

### Motility of HCMV-Infected Monocytes

Monocytes rapidly infiltrate into multiple organs following appropriate stimuli (Jakubzick et al., 2017; Patel et al., 2017) in a process that facilitates HCMV dissemination in these infected monocytes (Bentz et al., 2006; de Witte et al., 2008; Nogalski et al., 2011; Chan et al., 2012; Nikitina et al., 2018; Ayala-Nunez et al., 2019). Other viruses are also known to use this inherent monocyte ability. For example, ZIKV-infected monocytes with high level expression of adhesion molecules such as CD162, CD169, and CD43, show increased attachment to brain endothelium, which may promote ZIKV infection of neuronal cells (Ayala-Nunez et al., 2019). Monocytes infected with human immunodeficient virus (HIV) can penetrate the blood-brain barrier due to disruption and reduced expression of tight junction proteins such as ZO-1, occludin, and claudin in the barrier (Boven et al., 2000; Spindler and Hsu, 2012).

HCMV-infected monocytes can infiltrate a broad range of tissues, which in turn results in multiorgan pathogenesis. The migration of myeloid cells to specific sites is tightly regulated and determined by chemokines (Rossi and Zlotnik, 2000). However, it has been reported that expression of chemokine receptors such as CCR1, CCR2, CCR5, and CXCR4 is reduced on the surface of HCMV-infected monocytes, causing HCMV-infected monocytes to be less response to these chemokines (Frascaroli et al., 2006). Yet, overtime the HCMV-infected monocytes showed greater movement than PMA treated monocytes. This pathogenic motility was dependent on PI(3)K and the actin cytoskeleton as LY294002 (an inhibitor of PI(3)K) or cytochalasin D (the inhibitor of actin polymerization) treatment blocked motility (Smith et al., 2004a,b). In addition, the activation of PI(3)K via integrin and EGFR signaling induces the phosphorylation of paxillin, a key molecule of actin rearrangement, and upregulation of an actin-nucleator not usually associated with monocyte motility, N-WASP (Chan et al., 2009b; Nogalski et al., 2011). Collectively, the same signals that trigger differentiation also stimulate motility in a chemotaxis-independent manner, known as chemokinesis.

### Survival of HCMV-Infected Monocytes

Viability of blood monocytes is tightly regulated by apoptosis, which is triggered by a variety of death signals or diverse microenvironmental perturbations and caspase-mediated cell death processes (Galluzzi et al., 2012). Survival of infected monocytes is important for their differentiation, replication and persistence. Although these are discrete biological processes that are interrelated, ultimately similar pathways are involved in both processes. In serum-free medium, more than 70% of monocytes spontaneously undergo apoptosis and show the activation of caspase 3 within 16 h (Fahy et al., 1999). Due to the short half-life of monocytes, pathogens targeting and utilizing monocytes must have a “strategy” to extend their life span (Nikitina et al., 2018). HCMV-infected monocytes/macrophages can survive for long periods of times (weeks ~ years), which allows long-term viral production in infected cells (Wardley and Wilkinson, 1978; Nagra et al., 1993; Mistrikova et al., 1994; Radkowski et al., 2005; Psalla et al., 2006; Nikitina et al., 2018). HCMV-infected monocytes can survive for months-to-years as a result of subverting various cell death pathways (Smith et al., 2004a). HCMV glycoproteins interacting with cellular receptors activates anti-apoptotic molecules such as Bcl-2 family members to inhibit apoptosis (Chan et al., 2010; Collins-McMillen et al., 2015). In addition, a number of other cell death pathways are regulated by variety of viral gene products (reviewed in Collins-McMillen et al., 2018b). It is expected that once productive infection is initiated, in the context of viral lytic gene expression, these viral produced anti-apoptotic factors such as pUL36, pUL37x1, and pUL38 to name a few (reviewed in Collins-McMillen et al., 2018b) would play key roles in the extended survival of these infected macrophages. Collectively, HCMV-induced signaling controls the expression of anti-apoptotic molecules such as Mcl-1 and Bcl-2 and others, resulting in the long-term survival of HCMV-infected monocytes, which in turn allows differentiation to proceed.

### Mechanisms of Monocyte to Macrophage Differentiation During HCMV Infection

**Figure 2:**
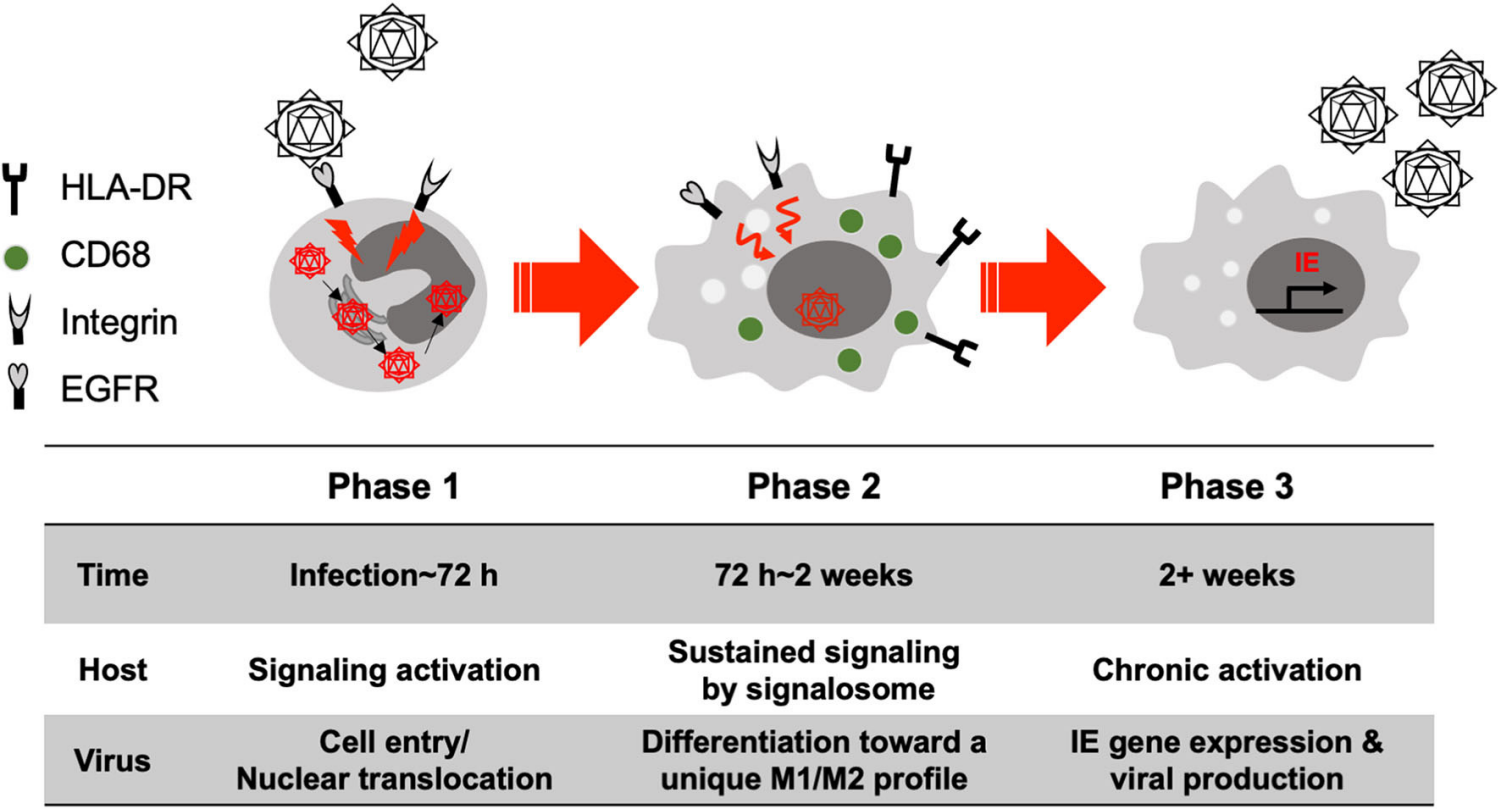
Simply model for the differentiation of primary HCMV-infected monocytes. We have loosely grouped the process of differentiation into 3 phases. Phase (1) Viral glycoproteins interact with cellular receptors during cell entry, which activates a variety of signaling cascade that in turn promote the early stages of differentiation (<72 h after infection). Phase (2) The activated signaling is sustained and continues to induce monocyte differentiation into a unique M1/M2 macrophage (72 h ~ 2 weeks after infection). Phase (3) Differentiated macrophages show chronic activation and long-term survival (months to years) and show expression of a full cascade of viral genes and production of infectious virions (> 2 weeks after infection).

Monocytes are considered a major vector or Trojan Horse for hematogenous dissemination of the virus following primary infection and upon reactivation from latency. Thus, there is a strong need to better understand the pathological consequences of their infection (Streblow and Nelson, 2003; Chan et al., 2012). Upon initial infection monocytes generate very little viral gene expression, with viral genes only being seen 2–3 weeks after infection and cellular differentiation (Ibanez et al., 1991; Smith et al., 2004a, 2010; Stevenson et al., 2014; Kim et al., 2016). These results suggest that cellular differentiation is required for the initiation of the expression of IE genes and viral replication (Ibanez et al., 1991; Smith et al., 2004a; Nogalski et al., 2011). For HCMV-induced monocyte to macrophage differentiation, we have broadly outlined three steps (Figure 2). (1) Receptor-ligand interactions and the initiation of differentiation, (2) Full differentiation with concomitant viral nuclear translocation and (3) IE gene expression and mature virus release from long-term infected macrophages. We will discuss each step during primary infection of HCMV in monocytes/macrophages below.

### HCMV-Induced Receptor-Ligand Signaling

**Figure 3:**
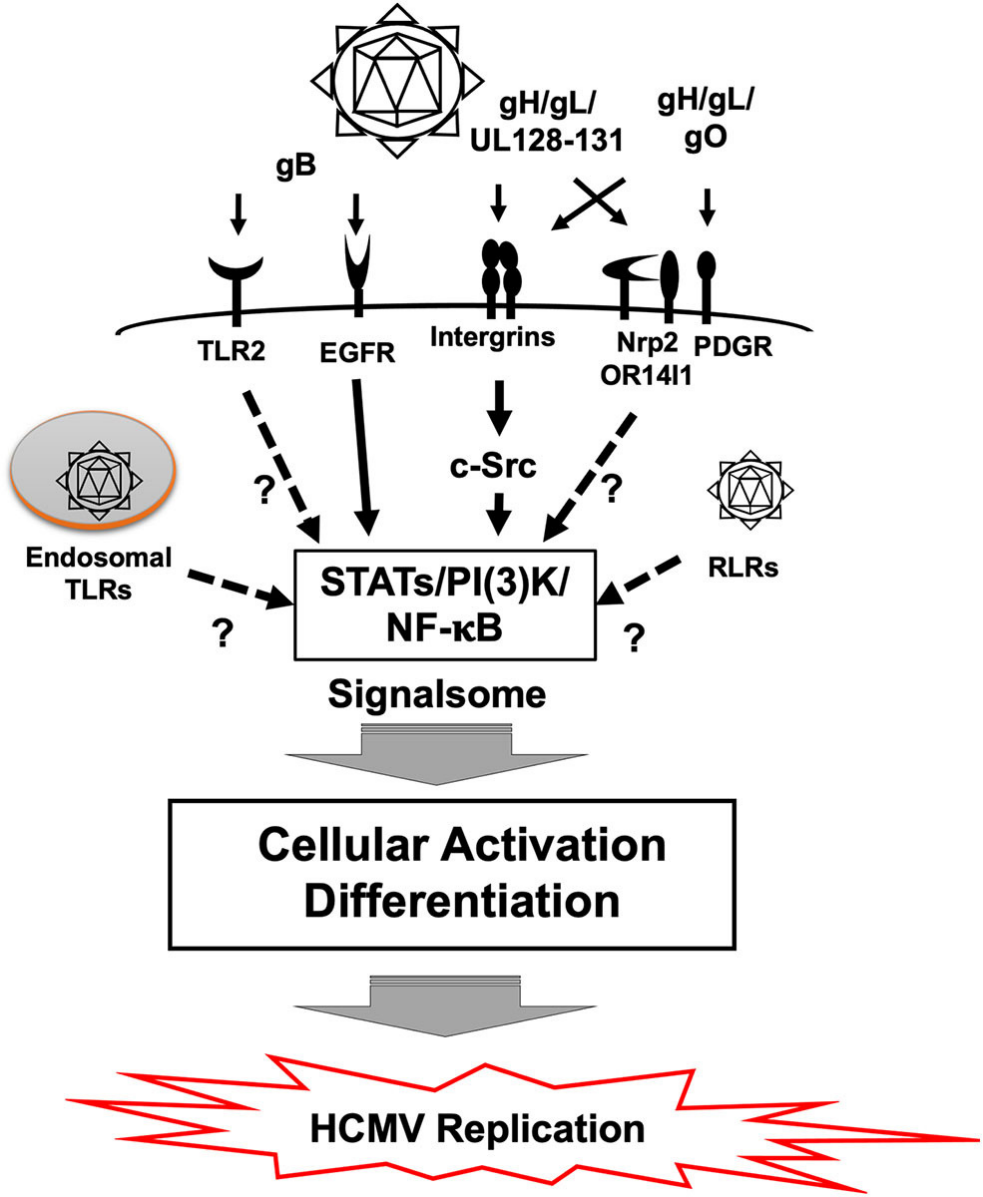
Signal transduction in monocytes during HCMV primary infection. Binding of gB to EGFR and the pentamer to β_1_ and β_3_ integrins activates downstream signaling pathways via the EGFR tyrosine kinase and c-Src, respectively. This gB and pentamer activation of monocytes creates a viral signalosome that leads the differentiation of monocytes-into-macrophages. Monocytes can also be activated by Toll-like receptors (TLRs) and RIG-I like receptors (RLR), which recognizes pathogen associated molecular patterns (PAMP). It is unclear how TLRs and RLRs affect differentiation of monocytes following HCMV infection. Other potential receptors also exist and how they influence monocyte infection is also unknown.

HCMV infection results in rapid cellular activation and early steps in the differentiation process of monocytes during the cell binding and entry phase via a pathogen associated molecular pattern (PAMP) independent manner (Boehme et al., 2006; Smith et al., 2007; Chan et al., 2009b; Yew et al., 2010, 2012; Nogalski et al., 2011, 2013; Kim et al., 2016; Collins-McMillen et al., 2017). Glycoproteins on the HCMV envelop engage several cellular receptors, including the epidermal growth factor receptor (EGFR), β_1_ and β_3_ integrins and heparan sulfate proteoglycans (Wang et al., 2003; Feire et al., 2004; Nogalski et al., 2013) (Figure 3). Other receptors have also been documented to bind to various viral glycoproteins (Soroceanu et al., 2008; Martinez-Martin et al., 2018; Xiaofei et al., 2019). However, it remains unclear the role that these receptors play during entry and signaling in monocytes. HCMV glycoprotein B (gB) and gH/gL/UL128-131 (the Pentamer), respectively, engages EGFR and β_1_/β_3_ integrins for viral entry, which induces the activation of PI(3)K and other signaling pathways (Chan et al., 2008, 2009a,b, 2012; Nogalski et al., 2013). The HCMV-induced signalosome following viral binding contributes to monocyte differentiation and motility, resulting in the induction of the unique M1/M2 macrophage phenotype (Ibanez et al., 1991; Smith et al., 2004a; Bentz et al., 2006; Chan et al., 2008).

Our data collectively supports that the interaction between viral envelop glycoproteins and cellular receptors leads to monocyte differentiation into macrophages (Ibanez et al., 1991; Smith et al., 2004a; Stevenson et al., 2014; Collins-McMillen et al., 2017). Furthermore, it does not appear that expression of *de novo* viral gene products is required for monocyte-to-macrophage differentiation because we have observed a delayed nuclear translocation of the viral genome until 3 days post-infection (Kim et al., 2016) and a lack of *de novo* IE gene expression until around 3 weeks post-infection, which is after cellular differentiation has occurred (Smith et al., 2004a; Nogalski et al., 2013). Furthermore, use of UV-inactivated HCMV also induces monocyte differentiation in a manner similar to that seen with live HCMV, indicating that ligand-receptor interaction without the expression of viral genes plays a pivotal role in the differentiation of HCMV infected-monocytes (Smith et al., 2004a). Lastly, through blockade of type I and II interferons, we also noted that these interferon pathways are not involved early in the infection process (Collins-McMillen et al., 2017).

HCMV engagement of cellular receptors initiates not only viral entry but also monocyte differentiation into macrophages. EGFR is required for viral entry via interaction with viral gB (Wang et al., 2003; Chan et al., 2009b) (Figure 3). A recent study showed that EGFR and integrin signaling are required for HCMV mediated monocyte differentiation (Smith et al., 2007; Chan et al., 2009b, 2012; Nogalski et al., 2013; Collins-McMillen et al., 2017). Pharmacological inhibition of EGFR with AG1478 (EGFR tyrosine kinase inhibitor), and/or PP2 (Src kinase inhibitor) significantly reduced phosphorylation at Y701 and S727 in signal transducer and activator of transcription 1 (STAT1), which in turn affected monocyte function and differentiation (Collins-McMillen et al., 2017). Also, knockdown and inhibition of STAT1 by siRNA and fludarabine (an inhibitor of STAT1 activation) further implicated receptor-ligand signal induced STAT1 in differentiation of monocytes into macrophages. Although STAT1 plays a key antiviral role during most infections, we argued in this study that phosphorylation and upregulation of STAT1 was used during HCMV infection to promote monocyte differentiation and that activation of the EGFR and integrin signaling pathways following viral binding initiated this process.

The viral genome is rapidly translocated into the nucleus of fibroblasts and epithelial/endothelial cells; however, in monocytes, there is an extended nuclear translocation and trafficking process in which the virus must move through the trans-golgi network and recycling endosomes before nuclear translocation occurs around 3 days post infection (Kim et al., 2016). Receptor-ligand signaling between integrins and the gH/gL/UL128-131-complex is required for viral entry, endosomal trafficking and nuclear translocation (Kim et al., 2016; Collins-McMillen et al., 2017). Pharmacological inhibition of β_1_ and β_3_ integrin induced signaling through c-Src reduced efficient viral entry, and showed that the viral particles that did enter the cell underwent rapid lysosomal degradation, further emphasizing the importance of early signaling (Nogalski et al., 2013; Kim et al., 2016). HCMV gB engages EGFR, as discussed above, and this engagement is also essential for viral entry and productive infection (Chan et al., 2009b); demonstrating that multiple signaling complexes exist that work in cooperation to drive the biological processes required for productive infection of monocytes/macrophages.

### PAMP-Dependent Differentiation

PAMPs are recognized by Pattern Recognition Receptors (PRRs) such as Toll-like receptors (TLRs) or the various intracellular biosensors such as Retinoic Acid Inducible Gene I (RIG-I), cyclic GMP-AMP synthase (cGAS), and Stimulator of Interferon Gene (STING) (Takeuchi and Akira, 2010; Li and Chen, 2018). This PRR/PAMP engagement induces inflammatory cytokines and activates many different immune response pathways. This PRR/PAMP engagement can also result in monocyte differentiation under some circumstances (Krutzik et al., 2005). HCMV components have been reported to be recognized by PRRs and to lead to the production of inflammatory cytokines (Figure 3). For example, purified gB interacts with TLR2 (Boehme et al., 2006). HCMV infection was also seen to induce the expression of IL-12 and TNF-α in THP-1 cells via TLR2 and 9, respectively (Yew et al., 2010). cGAS, a cytosolic DNA sensor, but not IFI16, recognizes HCMV and induces type I interferons (Type I IFNs), in endothelial cells (Lio et al., 2016). IFI16, however, can regulate HCMV replication in fibroblasts even though a relationship with Type I IFNs was not shown (Gariano et al., 2012). This PAMP dependent signaling can alter infection of some cell types and under some circumstances contribute to the differentiation of HCMV-infected monocytes, although more work needs to be undertaken to elucidate this process further. We have noted that human monocyte activation occurs in the absence of a robust IFN response (Collins-McMillen et al., 2017) and thus argue that glycoprotein-cell receptor engagement is the key event dictating monocyte differentiation following primary infection. We also suggest that perhaps the observed extended trafficking is, in part, a mechanism to avoid PRRs and the immune response to the virus (Kim et al., 2016).

### Transcriptional Changes During Differentiation

**Figure 4:**
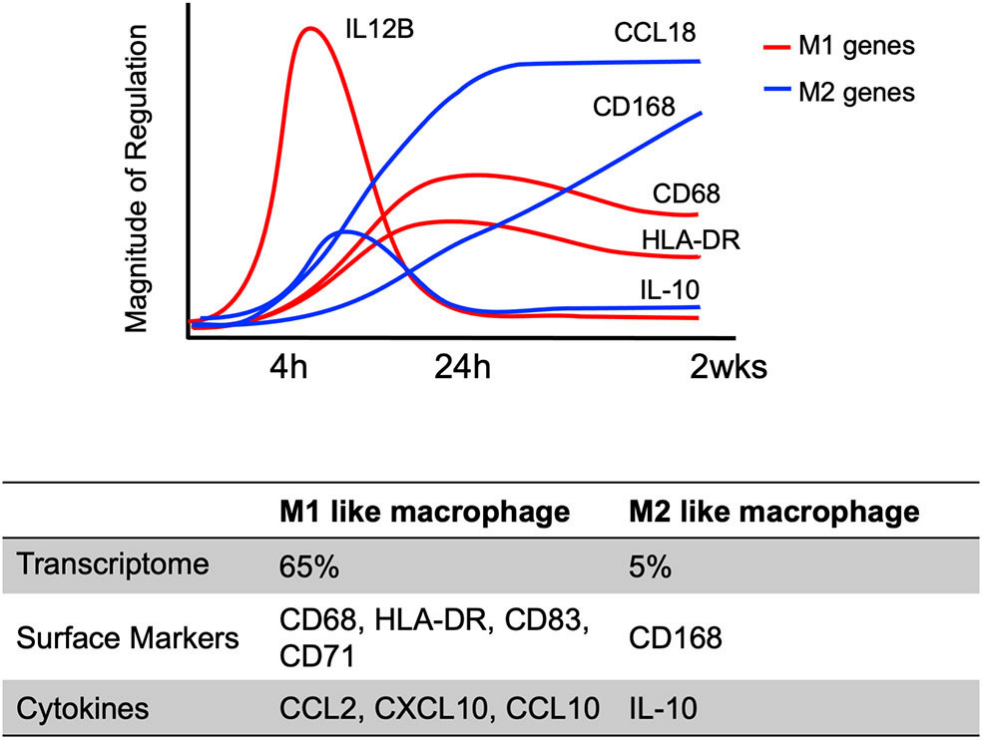
Kinetics of select M1/M2 genes in HCMV infected monocytes/macrophages. M1 and M2 macrophage related genes in HCMV-infected monocytes are differently expressed in a time dependent manner (4 h ~ 2 weeks after HCMV infection). The graph indicates the temporal changes in M1 (Red) or M2 (Blue) macrophage related gene expression patterns using a select few genes to emphasize the patterns of expression. The results are reanalyzed data of previous transcriptome analyses (Chan et al., 2008; Stevenson et al., 2014).

The initiation of differentiation occurs early, with significant transcriptional changes seen ~4–24 h after infection that is strongly induced by engagement of viral glycoproteins with various cellular receptors (Chan et al., 2008, 2009a; Stevenson et al., 2014) (Figure 4). For example, at 4 h post-infection, 65% of genes related to classic M1 macrophage polarization (inflammatory macrophage) are up-regulated in HCMV primary infected monocytes, while only 4% of genes associated with classic M2 macrophage polarization (resolving/wound healing macrophage) are upregulated (Chan et al., 2012). On the other hand, proteomic analysis of the secretome identified that a similar level of M1 (44%) and of M2 (33%) chemokines were produced. In addition, these early transcriptional changes were controlled by NF-κB and PI(3)K signaling events as treatment with pharmacological inhibitors of these pathways (Bay 11-7802; an inhibitor of NF-κB activity or LY294002; an inhibitor of PI(3)K activity) inhibited expression of 30–50% of M1 and 100% of M2 upregulated genes in infected monocytes. Interestingly, the cytokines induced during this time frame are monocyte trophic recruiting factors such as CCL2, CXCL10 and CCL15 as well as the anti-inflammatory cytokine IL-10 (Chan et al., 2008). Not only are there early transcriptional changes, but there are also long-term transcriptional changes in HCMV-infected monocytes. Transcriptional analysis showed a dynamic change in M1 and M2 related genes over 4 h ~ 2 weeks course of infection (Figure 4) (Chan et al., 2008; Stevenson et al., 2014). Viral homologs of human IL-10 (UL111A) have been reported to induce the differentiation of monocytes toward an M2 macrophage (Avdic et al., 2013, 2016), perhaps this IL-10 made prior to viral gene expression has a similar function. This data suggests HCMV infection promotes monocyte recruitment into tissues where upon differentiation occurs. The HCMV differentiated macrophage shows a unique M1/M2 phenotype and transcriptional and secretomic profiling suggests the virus creates a long term “perfect” macrophage for viral replication.

### Differentiation During Reactivation From Latency

CD34^+^ HPCs in the bone marrow are considered to be the major reservoir for HCMV latency (Streblow and Nelson, 2003; Cheng et al., 2017; Collins-McMillen et al., 2018a; Buehler et al., 2019). Monocytes discussed above likely carry virus into the bone marrow following primary infection to promote the establishment of latency (Chan et al., 2012). During reactivation from latency, the differentiation of CD34^+^ HPCs into monocytes is important for widespread viral dissemination (Sinclair, 2008; Smith et al., 2010; Crawford et al., 2018; Zhu et al., 2018; Hancock et al., 2020). Although the initiation of viral reactivation in CD34^+^ HPCs remains unresolved, it is known that a number of viral genes such as US28 and UL7 are involved in the differentiation of CD34^+^ HPC during reactivation (Crawford et al., 2018, 2019; Zhu et al., 2018). Other products such as UL138, LUNA are also involved in reactivation (Lee et al., 2015; Collins-McMillen et al., 2018a; Poole et al., 2018; Elder and Sinclair, 2019). We briefly discuss below those gene products that have been reported to be involved in cellular differentiation.

### US28

US28, a viral G protein-coupled receptor, is strongly associated with the differentiation of CD34^+^ HPCs into monocytes during reactivation (Vomaske et al., 2009a,b; Zhu et al., 2018; Crawford et al., 2019). US28 expression during latent-infection of CD34^+^ HPCs plays a key role in the maintenance of latency (Vomaske et al., 2009a; Poole et al., 2013; Crawford et al., 2015, 2019; Cheng et al., 2017; Krishna et al., 2019). A recent study showed that US28 is involved in the programming of CD34^+^ HPCs for a longer-life span, perhaps to better allow differentiation into monocytes or macrophages (Zhu et al., 2018). A US28 deficient mutant HCMV was unable to activate the cellular signaling transducer and activator of transcription 3 (STAT3) and failed to differentiate CD34^+^ HPCs into monocytes expressing CD14, CD11b, M-CSFR, and CD16 (Zhu et al., 2018). Furthermore, in a humanized mice model, a US28-Y16F mutant virus (a ligand binding mutant) was unable to maintain latency in CD34^+^ HPCs, which suggests that US28 ligand binding is required for the maintenance of HCMV latency in CD34^+^ HPCs (Vomaske et al., 2009a; Crawford et al., 2019)

### UL7

HCMV UL7 is a novel carcinoembryonic antigen-related cell adhesion molecule 1 (CEACAM-1)- like molecule, that is homologous to CD229, the signaling lymphocyte-activation molecule (SLAM) which is involved in leukocyte activation (MacManiman et al., 2014). It was reported that UL7 transfected myeloid cells, including immature dendritic cells, monocytes, and macrophage cell lines showed reduced production of pro-inflammatory cytokines such as IL-8, IL-6, and TNF with or without co-treatment with PMA or lipopolysaccharide (Engel et al., 2011). Importantly for myeloid cell differentiation, a recent study showed that UL7 induces CD34^+^ HPC and monocyte differentiation (Crawford et al., 2018). Furthermore, UL7 was shown to bind to the Flt3R and induce phosphorylation and activate downstream molecules including Akt and ERK1/2. This was the first report of an HCMV gene product acting like a stem cell-like factor.

### HCMV Replication in Differentiated Macrophages

The replication of HCMV in primary infected myeloid cells is closely related to their differentiation status (Chan et al., 2012; Stevenson et al., 2014). The same point holds true for these cells during reactivation of latent virus. The differentiation of monocytes, that are latently infected with HCMV, require differentiation of the cells into macrophages in order to show lytic gene expression (Taylor-Wiedeman et al., 1994; Soderberg-Naucler et al., 1997; Smith et al., 2010; Nogalski et al., 2013). A similar process occurs in seropositive individuals, as allogenic stimulation and differentiation was shown to be essential for viral replication in HCMV-infected monocytes isolated from a seropositive individual (Taylor-Wiedeman et al., 1994; Soderberg-Naucler et al., 1997; Smith et al., 2010). Allogenic stimulation induced the reactivation of viral replication, but also the differentiation of monocytes into macrophages expressing M1 polarized markers such as CD14, CD64, CD83, and HLA-DR. Interestingly, the study showed that allogenic stimulated monocytes also expressed a surface marker of dendritic cells—CD1a. Consistent with the result seen following allogenic stimulation of monocytes from seropositive individual, *in vitro* primary infected monocytes expressed CD68 and HLA-DR (Smith et al., 2004a). These primary infected HCMV infected monocytes also expressed the M1-specific markers, CD86 and CD71, and the M2-specific maker, CD163 (Stevenson et al., 2014). These initial phenotypic analyses are consistent with the data from various transcriptomes showing a unique M1/M2 phenotype in the differentiated macrophages, demonstrating that in many cases whether primary infection of monocytes, which results in differentiation to productively infected macrophages or reactivation and differentiation of that latently infected monocyte to a productively infected macrophages there seems to be similar phenotypic characteristics (even though molecular and biological differences exist). Interestingly, these infected allogenic stimulated monocytes/macrophages generated detectable levels of IE proteins at 4–5 days post reactivation and viral late proteins started to accumulate around 7 days post stimulation. Ultimately, it appears that differentiation is required and essential for HCMV infected monocytes to become productively infected macrophages and to efficiently produce infectious virions (Ibanez et al., 1991; Smith et al., 2004a; Stevenson et al., 2014).

## Future Perspective

A distinct combination of viral and cellular mechanisms orchestrates the differentiation of HCMV-infected myeloid cells. This differentiation of monocytes into tissue macrophages is a required step both in primary infection and in reactivating virus in monocytes. Since previous studies elucidated some of the responsible factors for the differentiation using advanced techniques and diverse models, we now need to focus on the specific factors driving the molecular and biological processes with an attention toward identifying the steps that could be blocked to mitigate disease. We also need to better characterize the differentiated macrophage in order to understand how HCMV manipulates the immune system of the host. Since monocytes differentiate into a diverse array of macrophages via various environmental factors *in vivo*, we need to define how HCMV controls specific points of differentiation using *ex vivo* or equivalent systems in order to better define mechanism involved and what the ideal virally infected long-term macrophage really “looks like.” Even though these questions are difficult to answer, these results would provide a new point for discussion of HCMV pathogenesis during primary infection and reactivation and the information would help promote the development of new drug targets that could be used to alleviate disease in immunocompromised patients.

## Author Contributions

All authors listed have made a substantial, direct and intellectual contribution to the work, and approved it for publication.

## Conflict of Interest

The authors declare that the research was conducted in the absence of any commercial or financial relationships that could be construed as a potential conflict of interest.

## References

[B1] Adland E. Klenerman P. Goulder P. Matthews P. C. (2015). Ongoing burden of disease and mortality from HIV/CMV coinfection in Africa in the antiretroviral therapy era. Front. Microbiol. 6:1016. 10.3389/fmicb.2015.01016 26441939 PMC4585099

[B2] Arandjelovic S. Ravichandran K. S. (2015). Phagocytosis of apoptotic cells in homeostasis. Nat. Immunol. 16, 907–917. 10.1038/ni.3253 26287597 PMC4826466

[B3] Arribas J. R. Storch G. A. Clifford D. B. Tselis A. C. (1996). Cytomegalovirus encephalitis. Ann. Intern. Med. 125, 577–587. 10.7326/0003-4819-125-7-199610010-00008 8815757

[B4] Avdic S. Cao J. Z. McSharry B. P. Clancy L. E. Brown R. Steain M.. (2013). Human cytomegalovirus interleukin-10 polarizes monocytes toward a deactivated M2c phenotype to repress host immune responses. J. Virol. 87, 10273–10282. 10.1128/JVI.00912-13 23864618 PMC3754025

[B5] Avdic S. McSharry B. P. Steain M. Poole E. Sinclair J. Abendroth A.. (2016). Human cytomegalovirus-encoded human interleukin-10 (IL-10) homolog amplifies its immunomodulatory potential by upregulating human IL-10 in monocytes. J. Virol. 90, 3819–3827. 10.1128/JVI.03066-15 26792743 PMC4810557

[B6] Ayala-Nunez N. V. Follain G. Delalande F. Hirschler A. Partiot E. Hale G. L.. (2019). Zika virus enhances monocyte adhesion and transmigration favoring viral dissemination to neural cells. Nat. Commun. 10:4430. 10.1038/s41467-019-12408-x 31562326 PMC6764950

[B7] Bentz G. L. Jarquin-Pardo M. Chan G. Smith M. S. Sinzger C. Yurochko A. D. (2006). Human cytomegalovirus (HCMV) infection of endothelial cells promotes naive monocyte extravasation and transfer of productive virus to enhance hematogenous dissemination of HCMV. J. Virol. 80, 11539–11555. 10.1128/JVI.01016-06 16987970 PMC1642592

[B8] Boeckh M. Nichols W. G. Papanicolaou G. Rubin R. Wingard J. R. Zaia J. (2003). Cytomegalovirus in hematopoietic stem cell transplant recipients: current status, known challenges, and future strategies. Biol. Blood Marrow Transplant. 9, 543–558. 10.1016/S1083-8791(03)00287-8 14506657

[B9] Boehme K. W. Guerrero M. Compton T. (2006). Human cytomegalovirus envelope glycoproteins B and H are necessary for TLR2 activation in permissive cells. J. Immunol. 177, 7094–7102. 10.4049/jimmunol.177.10.7094 17082626

[B10] Boven L. A. Middel J. Verhoef J. De Groot C. J. Nottet H. S. (2000). Monocyte infiltration is highly associated with loss of the tight junction protein zonula occludens in HIV-1-associated dementia. Neuropathol. Appl. Neurobiol. 26, 356–360. 10.1046/j.1365-2990.2000.00255.x 10931369

[B11] Britt W. J. (2018). Maternal immunity and the natural history of congenital human cytomegalovirus infection. Viruses 10:405. 10.3390/v10080405 30081449 PMC6116058

[B12] Buehler J. Carpenter E. Zeltzer S. Igarashi S. Rak M. Mikell I.. (2019). Host signaling and EGR1 transcriptional control of human cytomegalovirus replication and latency. PLoS Pathog. 15:e1008037. 10.1371/journal.ppat.1008037 31725811 PMC6855412

[B13] Chan G. Bivins-Smith E. R. Smith M. S. Smith P. M. Yurochko A. D. (2008). Transcriptome analysis reveals human cytomegalovirus reprograms monocyte differentiation toward an M1 macrophage. J. Immunol. 181, 698–711. 10.4049/jimmunol.181.1.698 18566437 PMC2614917

[B14] Chan G. Bivins-Smith E. R. Smith M. S. Yurochko A. D. (2009a). NF-kappaB and phosphatidylinositol 3-kinase activity mediates the HCMV-induced atypical M1/M2 polarization of monocytes. Virus Res. 144, 329–333. 10.1016/j.virusres.2009.04.026 19427341 PMC2736317

[B15] Chan G. Nogalski M. T. Bentz G. L. Smith M. S. Parmater A. Yurochko A. D. (2010). PI3K-dependent upregulation of Mcl-1 by human cytomegalovirus is mediated by epidermal growth factor receptor and inhibits apoptosis in short-lived monocytes. J. Immunol. 184, 3213–3222. 10.4049/jimmunol.0903025 20173022 PMC3743441

[B16] Chan G. Nogalski M. T. Stevenson E. V. Yurochko A. D. (2012). Human cytomegalovirus induction of a unique signalsome during viral entry into monocytes mediates distinct functional changes: a strategy for viral dissemination. J. Leukoc. Biol. 92, 743–752. 10.1189/jlb.0112040 22715139 PMC3441319

[B17] Chan G. Nogalski M. T. Yurochko A. D. (2009b). Activation of EGFR on monocytes is required for human cytomegalovirus entry and mediates cellular motility. Proc. Natl. Acad. Sci. U.S.A. 106, 22369–22374. 10.1073/pnas.0908787106 20018733 PMC2799688

[B18] Cheng S. Caviness K. Buehler J. Smithey M. Nikolich-Zugich J. Goodrum F. (2017). Transcriptome-wide characterization of human cytomegalovirus in natural infection and experimental latency. Proc. Natl. Acad. Sci. U.S.A. 114, E10586–E10595. 10.1073/pnas.1710522114 29158406 PMC5724264

[B19] Collins-McMillen D. Buehler J. Peppenelli M. Goodrum F. (2018a). Molecular determinants and the regulation of human cytomegalovirus latency and reactivation. Viruses 10:8. 10.3390/v10080444 30127257 PMC6116278

[B20] Collins-McMillen D. Chesnokova L. Lee B. J. Fulkerson H. L. Brooks R. Mosher B. S.. (2018b). HCMV infection and apoptosis: how do monocytes survive HCMV infection? Viruses 10:10. 10.3390/v10100533 30274264 PMC6213175

[B21] Collins-McMillen D. Kim J. H. Nogalski M. T. Stevenson E. V. Chan G. C. Caskey J. R.. (2015). Human cytomegalovirus promotes survival of infected monocytes via a distinct temporal regulation of cellular Bcl-2 family proteins. J. Virol. 90, 2356–2371. 10.1128/JVI.01994-15 26676786 PMC4810730

[B22] Collins-McMillen D. Stevenson E. V. Kim J. H. Lee B. J. Cieply S. J. Nogalski M. T.. (2017). Human cytomegalovirus utilizes a nontraditional signal transducer and activator of transcription 1 activation cascade via signaling through epidermal growth factor receptor and integrins to efficiently promote the motility, differentiation, and polarization of infected monocytes. J. Virol. 91:24. 10.1128/JVI.00622-17 29021395 PMC5709601

[B23] Crawford L. B. Caposio P. Kreklywich C. Pham A. H. Hancock M. H. Jones T. A.. (2019). Human cytomegalovirus US28 ligand binding activity is required for latency in CD34(+) hematopoietic progenitor cells and humanized NSG mice. MBio 10:19. 10.1128/mBio.01889-19 31431555 PMC6703429

[B24] Crawford L. B. Kim J. H. Collins-McMillen D. Lee B. J. Landais I. Held C.. (2018). Human cytomegalovirus encodes a novel FLT3 receptor ligand necessary for hematopoietic cell differentiation and viral reactivation. MBio 9:2. 10.1128/mBio.00682-18 29691342 PMC5915732

[B25] Crawford L. B. Streblow D. N. Hakki M. Nelson J. A. Caposio P. (2015). Humanized mouse models of human cytomegalovirus infection. Curr. Opin. Virol. 13, 86–92. 10.1016/j.coviro.2015.06.006 26118890 PMC4599783

[B26] de Witte L. de Vries R. D. van der Vlist M. Yuksel S. Litjens M. de Swart R. L.. (2008). DC-SIGN and CD150 have distinct roles in transmission of measles virus from dendritic cells to T-lymphocytes. PLoS Pathog. 4:e1000049. 10.1371/journal.ppat.1000049 18421379 PMC2277461

[B27] Elder E. Sinclair J. (2019). HCMV latency: what regulates the regulators? Med. Microbiol. Immunol. 208, 431–438. 10.1007/s00430-019-00581-1 30761409 PMC6647427

[B28] Engel P. Perez-Carmona N. Alba M. M. Robertson K. Ghazal P. Angulo A. (2011). Human cytomegalovirus UL7, a homologue of the SLAM-family receptor CD229, impairs cytokine production. Immunol. Cell Biol. 89, 753–766. 10.1038/icb.2011.55 21670740

[B29] Fahy R. J. Doseff A. I. Wewers M. D. (1999). Spontaneous human monocyte apoptosis utilizes a caspase-3-dependent pathway that is blocked by endotoxin and is independent of caspase-1. J. Immunol. 163, 1755–1762. 10438906

[B30] Feire A. L. Koss H. Compton T. (2004). Cellular integrins function as entry receptors for human cytomegalovirus via a highly conserved disintegrin-like domain. Proc. Natl. Acad. Sci. U.S.A. 101, 15470–15475. 10.1073/pnas.0406821101 15494436 PMC524452

[B31] Fonseca Brito L. Brune W. Stahl F. R. (2019). Cytomegalovirus (CMV) pneumonitis: cell tropism, inflammation, and immunity. Int. J. Mol. Sci. 20:16. 10.3390/ijms20163865 31398860 PMC6719013

[B32] Foo S. S. Chen W. Chan Y. Bowman J. W. Chang L. C. Choi Y.. (2017). Asian Zika virus strains target CD14(+) blood monocytes and induce M2-skewed immunosuppression during pregnancy. Nat. Microbiol. 2, 1558–1570. 10.1038/s41564-017-0016-3 28827581 PMC5678934

[B33] Frascaroli G. Varani S. Moepps B. Sinzger C. Landini M. P. Mertens T. (2006). Human cytomegalovirus subverts the functions of monocytes, impairing chemokine-mediated migration and leukocyte recruitment. J. Virol. 80, 7578–7589. 10.1128/JVI.02421-05 16840337 PMC1563711

[B34] Gabrielli L. Bonasoni M. P. Santini D. Piccirilli G. Chiereghin A. Petrisli E.. (2012). Congenital cytomegalovirus infection: patterns of fetal brain damage. Clin. Microbiol. Infect. 18, E419–E427. 10.1111/j.1469-0691.2012.03983.x 22882294

[B35] Galluzzi L. Vitale I. Abrams J. M. Alnemri E. S. Baehrecke E. H. Blagosklonny M. V.. (2012). Molecular definitions of cell death subroutines: recommendations of the Nomenclature Committee on Cell Death 2012. Cell Death Differ. 19, 107–120. 10.1038/cdd.2011.96 21760595 PMC3252826

[B36] Gariano G. R. Dell'Oste V. Bronzini M. Gatti D. Luganini A. De Andrea M.. (2012). The intracellular DNA sensor IFI16 gene acts as restriction factor for human cytomegalovirus replication. PLoS Pathog. 8:e1002498. 10.1371/journal.ppat.1002498 22291595 PMC3266931

[B37] Garrido E. Carrera E. Manzano R. Lopez-Sanroman A. (2013). Clinical significance of cytomegalovirus infection in patients with inflammatory bowel disease. World J. Gastroenterol. 19, 17–25. 10.3748/wjg.v19.i1.17 23326158 PMC3545225

[B38] Gilbert C. Boivin G. (2005). Human cytomegalovirus resistance to antiviral drugs. Antimicrob. Agents Chemother. 49, 873–883. 10.1128/AAC.49.3.873-883.2005 15728878 PMC549271

[B39] Goodrum F. (2016). Human cytomegalovirus latency: approaching the gordian knot. Annu. Rev. Virol. 3, 333–357. 10.1146/annurev-virology-110615-042422 27501258 PMC5514425

[B40] Goudot C. Coillard A. Villani A. C. Gueguen P. Cros A. Sarkizova S.. (2017). Aryl hydrocarbon receptor controls monocyte differentiation into dendritic cells versus macrophages. Immunity 47, 582–596. 10.1016/j.immuni.2017.08.016 28930664

[B41] Gredmark S. Britt W. B. Xie X. Lindbom L. Soderberg-Naucler C. (2004a). Human cytomegalovirus induces inhibition of macrophage differentiation by binding to human aminopeptidase N/CD13. J. Immunol. 173, 4897–4907. 10.4049/jimmunol.173.8.4897 15470031

[B42] Gredmark S. Soderberg-Naucler C. (2003). Human cytomegalovirus inhibits differentiation of monocytes into dendritic cells with the consequence of depressed immunological functions. J. Virol. 77, 10943–10956. 10.1128/JVI.77.20.10943-10956.2003 14512544 PMC224957

[B43] Gredmark S. Tilburgs T. Soderberg-Naucler C. (2004b). Human cytomegalovirus inhibits cytokine-induced macrophage differentiation. J. Virol. 78, 10378–10389. 10.1128/JVI.78.19.10378-10389.2004 15367604 PMC516431

[B44] Hancock M. H. Crawford L. B. Pham A. H. Mitchell J. Struthers H. M. Yurochko A. D.. (2020). Human cytomegalovirus miRNAs regulate TGF-beta to mediate myelosuppression while maintaining viral latency in CD34(+) hematopoietic progenitor cells. Cell Host Microbe 27, 104–114. 10.1016/j.chom.2019.11.013 31866424 PMC6952548

[B45] Heiden D. Ford N. Wilson D. Rodriguez W. R. Margolis T. Janssens B.. (2007). Cytomegalovirus retinitis: the neglected disease of the AIDS pandemic. PLoS Med. 4:e334. 10.1371/journal.pmed.0040334 18052600 PMC2100142

[B46] Hou W. Gibbs J. S. Lu X. Brooke C. B. Roy D. Modlin R. L.. (2012). Viral infection triggers rapid differentiation of human blood monocytes into dendritic cells. Blood 119, 3128–3131. 10.1182/blood-2011-09-379479 22310910 PMC3321872

[B47] Hume D. A. Irvine K. M. Pridans C. (2019). The mononuclear phagocyte system: the relationship between monocytes and macrophages. Trends Immunol. 40, 98–112. 10.1016/j.it.2018.11.007 30579704

[B48] Ibanez C. E. Schrier R. Ghazal P. Wiley C. Nelson J. A. (1991). Human cytomegalovirus productively infects primary differentiated macrophages. J. Virol. 65, 6581–6588. 10.1128/JVI.65.12.6581-6588.1991 1658363 PMC250717

[B49] Jakubzick C. Gautier E. L. Gibbings S. L. Sojka D. K. Schlitzer A. Johnson T. E.. (2013). Minimal differentiation of classical monocytes as they survey steady-state tissues and transport antigen to lymph nodes. Immunity 39, 599–610. 10.1016/j.immuni.2013.08.007 24012416 PMC3820017

[B50] Jakubzick C. V. Randolph G. J. Henson P. M. (2017). Monocyte differentiation and antigen-presenting functions. Nat. Rev. Immunol. 17, 349–362. 10.1038/nri.2017.28 28436425

[B51] Kim J. H. Collins-McMillen D. Caposio P. Yurochko A. D. (2016). Viral binding-induced signaling drives a unique and extended intracellular trafficking pattern during infection of primary monocytes. Proc. Natl. Acad. Sci. U.S.A. 113, 8819–8824. 10.1073/pnas.1604317113 27432979 PMC4978277

[B52] Krishna B. A. Humby M. S. Miller W. E. O'Connor C. M. (2019). Human cytomegalovirus G protein-coupled receptor US28 promotes latency by attenuating c-fos. Proc. Natl. Acad. Sci. U.S.A. 116, 1755–1764. 10.1073/pnas.1816933116 30647114 PMC6358704

[B53] Krutzik S. R. Tan B. Li H. Ochoa M. T. Liu P. T. Sharfstein S. E.. (2005). TLR activation triggers the rapid differentiation of monocytes into macrophages and dendritic cells. Nat. Med. 11, 653–660. 10.1038/nm1246 15880118 PMC1409736

[B54] Lanzieri T. M. Chung W. Flores M. Blum P. Caviness A. C. Bialek S. R.. (2017). Hearing loss in children with asymptomatic congenital cytomegalovirus infection. Pediatrics 139:3. 10.1542/peds.2016-2610 28209771 PMC5330400

[B55] Lee S. H. Albright E. R. Lee J. H. Jacobs D. Kalejta R. F. (2015). Cellular defense against latent colonization foiled by human cytomegalovirus UL138 protein. Sci. Adv. 1:e1501164. 10.1126/sciadv.1501164 26702450 PMC4681346

[B56] Li T. Chen Z. J. (2018). The cGAS-cGAMP-STING pathway connects DNA damage to inflammation, senescence, and cancer. J. Exp. Med. 215, 1287–1299. 10.1084/jem.20180139 29622565 PMC5940270

[B57] Lio C. W. McDonald B. Takahashi M. Dhanwani R. Sharma N. Huang J.. (2016). cGAS-STING signaling regulates initial innate control of cytomegalovirus infection. J. Virol. 90, 7789–7797. 10.1128/JVI.01040-16 27334590 PMC4988162

[B58] Maciejewski J. P. Bruening E. E. Donahue R. E. Sellers S. E. Carter C. Young N. S.. (1993). Infection of mononucleated phagocytes with human cytomegalovirus. Virology 195, 327–336. 10.1006/viro.1993.1383 8393230

[B59] MacManiman J. D. Meuser A. Botto S. Smith P. P. Liu F. Jarvis M. A.. (2014). Human cytomegalovirus-encoded pUL7 is a novel CEACAM1-like molecule responsible for promotion of angiogenesis. mBio 5:e02035. 10.1128/mBio.02035-14 25352622 PMC4217178

[B60] Martinez-Martin N. Marcandalli J. Huang C. S. Arthur C. P. Perotti M. Foglierini M.. (2018). An unbiased screen for human cytomegalovirus identifies neuropilin-2 as a central viral receptor. Cell 174, 1158–1171. 10.1016/j.cell.2018.06.028 30057110

[B61] Mendelson M. Monard S. Sissons P. Sinclair J. (1996). Detection of endogenous human cytomegalovirus in CD34+ bone marrow progenitors. J. Gen. Virol. 77, 3099–3102. 10.1099/0022-1317-77-12-3099 9000102

[B62] Mistrikova J. Remenova A. Lesso J. Stancekova M. (1994). Replication and persistence of murine herpesvirus 72 in lymphatic system and peripheral blood mononuclear cells of Balb/C mice. Acta Virol. 38, 151–156. 7817896

[B63] Nagra R. M. Wong P. K. Wiley C. A. (1993). Expression of major histocompatibility complex antigens and serum neutralizing antibody in murine retroviral encephalitis. J. Neuropathol. Exp. Neurol. 52, 163–173. 10.1097/00005072-199303000-00009 8382732

[B64] Nikitina E. Larionova I. Choinzonov E. Kzhyshkowska J. (2018). Monocytes and Macrophages as viral targets and reservoirs. Int. J. Mol. Sci. 19:9. 10.3390/ijms19092821 30231586 PMC6163364

[B65] Nogalski M. T. Chan G. Stevenson E. V. Gray S. Yurochko A. D. (2011). Human cytomegalovirus-regulated paxillin in monocytes links cellular pathogenic motility to the process of viral entry. J. Virol. 85, 1360–1369. 10.1128/JVI.02090-10 21084488 PMC3020497

[B66] Nogalski M. T. Chan G. C. Stevenson E. V. Collins-McMillen D. K. Yurochko A. D. (2013). The HCMV gH/gL/UL128-131 complex triggers the specific cellular activation required for efficient viral internalization into target monocytes. PLoS Pathog. 9:e1003463. 10.1371/journal.ppat.1003463 23853586 PMC3708883

[B67] Noriega V. M. Haye K. K. Kraus T. A. Kowalsky S. R. Ge Y. Moran T. M.. (2014). Human cytomegalovirus modulates monocyte-mediated innate immune responses during short-term experimental latency *in vitro*. J. Virol. 88, 9391–9405. 10.1128/JVI.00934-14 24920803 PMC4136239

[B68] Patel A. A. Zhang Y. Fullerton J. N. Boelen L. Rongvaux A. Maini A. A.. (2017). The fate and lifespan of human monocyte subsets in steady state and systemic inflammation. J. Exp. Med. 214, 1913–1923. 10.1084/jem.20170355 28606987 PMC5502436

[B69] Poole E. Walther A. Raven K. Benedict C. A. Mason G. M. Sinclair J. (2013). The myeloid transcription factor GATA-2 regulates the viral UL144 gene during human cytomegalovirus latency in an isolate-specific manner. J. Virol. 87, 4261–4271. 10.1128/JVI.03497-12 23365437 PMC3624344

[B70] Poole E. L. Kew V. G. Lau J. C. H. Murray M. J. Stamminger T. Sinclair J. H.. (2018). A virally encoded DeSUMOylase activity is required for cytomegalovirus reactivation from latency. Cell. Rep. 24, 594–606. 10.1016/j.celrep.2018.06.048 30021158 PMC6077246

[B71] Psalla D. Psychas V. Spyrou V. Billinis C. Papaioannou N. Vlemmas I. (2006). Pathogenesis of experimental encephalomyocarditis: a histopathological, immunohistochemical and virological study in mice. J. Comp. Pathol. 135, 142–145. 10.1016/j.jcpa.2006.04.003 16952370

[B72] Radkowski M. Gallegos-Orozco J. F. Jablonska J. Colby T. V. Walewska-Zielecka B. Kubicka J.. (2005). Persistence of hepatitis C virus in patients successfully treated for chronic hepatitis C. Hepatology 41, 106–114. 10.1002/hep.20518 15619235

[B73] Ramanan P. Razonable R. R. (2013). Cytomegalovirus infections in solid organ transplantation: a review. Infect. Chemother. 45, 260–271. 10.3947/ic.2013.45.3.260 24396627 PMC3848521

[B74] Rodero M. P. Poupel L. Loyher P. L. Hamon P. Licata F. Pessel C.. (2015). Immune surveillance of the lung by migrating tissue monocytes. Elife 4:e07847. 10.7554/eLife.07847 26167653 PMC4521583

[B75] Rossi D. Zlotnik A. (2000). The biology of chemokines and their receptors. Annu. Rev. Immunol. 18, 217–242. 10.1146/annurev.immunol.18.1.217 10837058

[B76] Serbina N. V. Jia T. Hohl T. M. Pamer E. G. (2008). Monocyte-mediated defense against microbial pathogens. Annu. Rev. Immunol. 26, 421–452. 10.1146/annurev.immunol.26.021607.090326 18303997 PMC2921669

[B77] Sinclair J. (2008). Human cytomegalovirus: Latency and reactivation in the myeloid lineage. J. Clin. Virol. 41, 180–185. 10.1016/j.jcv.2007.11.014 18164651

[B78] Sinzger C. Digel M. Jahn G. (2008). Cytomegalovirus cell tropism. Curr. Top. Microbiol. Immunol. 325, 63–83. 10.1007/978-3-540-77349-8_4 18637500

[B79] Sinzger C. Jahn G. (1996). Human cytomegalovirus cell tropism and pathogenesis. Intervirology 39, 302–319. 10.1159/000150502 9130041

[B80] Smith M. S. Bentz G. L. Alexander J. S. Yurochko A. D. (2004a). Human cytomegalovirus induces monocyte differentiation and migration as a strategy for dissemination and persistence. J. Virol. 78, 4444–4453. 10.1128/JVI.78.9.4444-4453.2004 15078925 PMC387677

[B81] Smith M. S. Bentz G. L. Smith P. M. Bivins E. R. Yurochko A. D. (2004b). HCMV activates PI(3)K in monocytes and promotes monocyte motility and transendothelial migration in a PI(3)K-dependent manner. J. Leukoc. Biol. 76, 65–76. 10.1189/jlb.1203621 15107461

[B82] Smith M. S. Bivins-Smith E. R. Tilley A. M. Bentz G. L. Chan G. Minard J.. (2007). Roles of phosphatidylinositol 3-kinase and NF-kappaB in human cytomegalovirus-mediated monocyte diapedesis and adhesion: strategy for viral persistence. J. Virol. 81, 7683–7694. 10.1128/JVI.02839-06 17507481 PMC1933358

[B83] Smith M. S. Goldman D. C. Bailey A. S. Pfaffle D. L. Kreklywich C. N. Spencer D. B.. (2010). Granulocyte-colony stimulating factor reactivates human cytomegalovirus in a latently infected humanized mouse model. Cell Host Microbe 8, 284–291. 10.1016/j.chom.2010.08.001 20833379 PMC2945885

[B84] Soderberg-Naucler C. Fish K. N. Nelson J. A. (1997). Reactivation of latent human cytomegalovirus by allogeneic stimulation of blood cells from healthy donors. Cell 91, 119–126. 10.1016/S0092-8674(01)80014-3 9335340

[B85] Soroceanu L. Akhavan A. Cobbs C. S. (2008). Platelet-derived growth factor-alpha receptor activation is required for human cytomegalovirus infection. Nature 455, 391–395. 10.1038/nature07209 18701889

[B86] Spindler K. R. Hsu T. H. (2012). Viral disruption of the blood-brain barrier. Trends Microbiol. 20, 282–290. 10.1016/j.tim.2012.03.009 22564250 PMC3367119

[B87] Stern L. Withers B. Avdic S. Gottlieb D. Abendroth A. Blyth E.. (2019). Human cytomegalovirus latency and reactivation in allogeneic hematopoietic stem cell transplant recipients. Front. Microbiol. 10:1186. 10.3389/fmicb.2019.01186 31191499 PMC6546901

[B88] Stevenson E. V. Collins-McMillen D. Kim J. H. Cieply S. J. Bentz G. L. Yurochko A. D. (2014). HCMV reprogramming of infected monocyte survival and differentiation: a Goldilocks phenomenon. Viruses 6, 782–807. 10.3390/v6020782 24531335 PMC3939482

[B89] Streblow D. N. Nelson J. A. (2003). Models of HCMV latency and reactivation. Trends Microbiol. 11, 293–295. 10.1016/S0966-842X(03)00149-5 12875809

[B90] Takeuchi O. Akira S. (2010). Pattern recognition receptors and inflammation. Cell 140, 805–820. 10.1016/j.cell.2010.01.022 20303872

[B91] Taylor-Wiedeman J. Sissons J. G. Borysiewicz L. K. Sinclair J. H. (1991). Monocytes are a major site of persistence of human cytomegalovirus in peripheral blood mononuclear cells. J. Gen. Virol. 72, 2059–2064. 10.1099/0022-1317-72-9-2059 1654370

[B92] Taylor-Wiedeman J. Sissons P. Sinclair J. (1994). Induction of endogenous human cytomegalovirus gene expression after differentiation of monocytes from healthy carriers. J. Virol. 68, 1597–1604. 10.1128/JVI.68.3.1597-1604.1994 8107221 PMC236617

[B93] Varol C. Mildner A. Jung S. (2015). Macrophages: development and tissue specialization. Annu. Rev. Immunol. 33, 643–675. 10.1146/annurev-immunol-032414-112220 25861979

[B94] Vomaske J. Melnychuk R. M. Smith P. P. Powell J. Hall L. DeFilippis V.. (2009a). Differential ligand binding to a human cytomegalovirus chemokine receptor determines cell type-specific motility. PLoS Pathog. 5:e1000304. 10.1371/journal.ppat.1000304 19229316 PMC2637432

[B95] Vomaske J. Nelson J. A. Streblow D. N. (2009b). Human Cytomegalovirus US28: a functionally selective chemokine binding receptor. Infect. Disord. Drug Targets 9, 548–556. 10.2174/187152609789105696 19594424 PMC3496389

[B96] Wang X. Huong S. M. Chiu M. L. Raab-Traub N. Huang E. S. (2003). Epidermal growth factor receptor is a cellular receptor for human cytomegalovirus. Nature 424, 456–461. 10.1038/nature01818 12879076

[B97] Wardley R. C. Wilkinson P. J. (1978). The growth of virulent African swine fever virus in pig monocytes and macrophages. J. Gen. Virol. 38, 183–186. 10.1099/0022-1317-38-1-183 340610

[B98] Wills M. R. Poole E. Lau B. Krishna B. Sinclair J. H. (2015). The immunology of human cytomegalovirus latency: could latent infection be cleared by novel immunotherapeutic strategies? Cell Mol. Immunol. 12, 128–138. 10.1038/cmi.2014.75 25132454 PMC4654298

[B99] Xiaofei E. Meraner P. Lu P. Perreira J. M. Aker A. M. McDougall W. M.. (2019). OR14I1 is a receptor for the human cytomegalovirus pentameric complex and defines viral epithelial cell tropism. Proc. Natl. Acad. Sci. U.S.A. 116, 7043–7052. 10.1073/pnas.1814850116 30894498 PMC6452726

[B100] Yamamoto A. Y. Mussi-Pinhata M. M. Isaac Mde L. Amaral F. R. Carvalheiro C. G. Aragon D. C.. (2011). Congenital cytomegalovirus infection as a cause of sensorineural hearing loss in a highly immune population. Pediatr. Infect. Dis. J. 30, 1043–1046. 10.1097/INF.0b013e31822d9640 21814153 PMC3222783

[B101] Yew K. H. Carpenter C. Duncan R. S. Harrison C. J. (2012). Human cytomegalovirus induces TLR4 signaling components in monocytes altering TIRAP, TRAM and downstream interferon-beta and TNF-alpha expression. PLoS ONE 7:e44500. 10.1371/journal.pone.0044500 22970235 PMC3436894

[B102] Yew K. H. Carsten B. Harrison C. (2010). Scavenger receptor A1 is required for sensing HCMV by endosomal TLR-3/-9 in monocytic THP-1 cells. Mol. Immunol. 47, 883–893. 10.1016/j.molimm.2009.10.009 19914718

[B103] Yi H. Zhang Y. Yang X. Li M. Hu H. Xiong J.. (2020). Hepatitis B core antigen impairs the polarization while promoting the production of inflammatory cytokines of M2 macrophages via the TLR2 pathway. Front. Immunol. 11:535. 10.3389/fimmu.2020.00535 32292408 PMC7118225

[B104] Yona S. Gordon S. (2015). From the reticuloendothelial to mononuclear phagocyte system - the unaccounted years. Front. Immunol. 6:328. 10.3389/fimmu.2015.00328 26191061 PMC4486871

[B105] Zhou Y. F. Leon M. B. Waclawiw M. A. Popma J. J. Yu Z. X. Finkel T.. (1996). Association between prior cytomegalovirus infection and the risk of restenosis after coronary atherectomy. N. Engl. J. Med. 335, 624–630. 10.1056/NEJM199608293350903 8687516

[B106] Zhu D. Pan C. Sheng J. Liang H. Bian Z. Liu Y.. (2018). Human cytomegalovirus reprogrammes haematopoietic progenitor cells into immunosuppressive monocytes to achieve latency. Nat. Microbiol. 3, 503–513. 10.1038/s41564-018-0131-9 29588542 PMC6537872

[B107] Zuhair M. Smit G. S. A. Wallis G. Jabbar F. Smith C. Devleesschauwer B.. (2019). Estimation of the worldwide seroprevalence of cytomegalovirus: a systematic review and meta-analysis. Rev. Med. Virol. 29:e2034. 10.1002/rmv.2034 30706584

